# Comment on: incidence of blindness in a population of rheumatic patients treated with hydroxychloroquine: reply

**DOI:** 10.1093/rap/rkz038

**Published:** 2019-10-30

**Authors:** Dilpreet K Singh, Leila Muhieddine, Douglas Einstadter, Stanley Ballou

**Affiliations:** MetroHealth Medical Center, Case Western Reserve University, Cleveland, OH, USA

Sir, we thank Pareek *et al.* [1] for their comments and interest in our article [2]. In response to their question, 6 of 31 (19.4%) patients with eye disease had diabetes mellitus, and 454 of 2836 (16.0%) without eye disease had diabetes mellitus. Note that these percentages are not significantly different (*P*-value = 0.61, χ^2^ test). We are unable to determine the mean duration of diabetes mellitus, because we have only the date when the diagnosis was entered onto the problem list, not the date when the condition was first diagnosed; it might be the same, but we are unable to be certain, particularly for those patients who may have been diagnosed with diabetes mellitus years before their initial visit to us.

Given that our original intent was to record the prevalence of blindness and HCQ maculopathy in a large cohort of patients on long-term therapy [2], we did not collect the large volume of detailed measures that would have permitted a much more thorough analysis of the potential importance of co-morbidities, such as diabetes mellitus and hypertension, and CS use on retinal toxicity with HCQ exposure. Unfortunately, given that the study has closed, we are unable to obtain additional data to determine the incidence of non-proliferative diabetic retinopathy in our population or further data on the patients with type 2 diabetes. We agree with Pareek *et al.* [1] regarding the potential importance of co-morbidities. Further study is needed. We appreciate their thoughtful comments.

*Funding*: No specific funding was received from any funding bodies in the public, commercial or not-for-profit sectors to carry out the work described in this manuscript.

*Disclosure statement*: The authors have declared no conflicts of interest.
